# Clinical application of the fracture risk assessment tool in the general population and its correlation with bone turnover markers

**DOI:** 10.3389/fphar.2022.1013483

**Published:** 2023-01-10

**Authors:** Zhi Yang, Shu Xuan, Weihong Li, Wan Hu, Ping Tu, Peng Duan

**Affiliations:** ^1^ Department of Endocrinology and Metabolism, The Third Hospital of Nanchang, Nanchang, China; ^2^ School of Physical Education, Liaoning Normal University, Dalian, China

**Keywords:** osteoporotic fracture, fracture risk assessment tool, bone mineral density, bone turnover markers, the urban areas, the urban-rural fringe areas

## Abstract

**Objective:** To compare the risk of osteoporotic fractures between the urban and urban-rural fringe populations in southern China and to explore the effect of bone turnover markers on fracture risk.

**Methods:** Epidemiological investigations were conducted in the urban and urban-rural fringe areas of southern China in June 2018. Residents aged 40 years and over who signed informed consent forms were included. Physical examination and questionnaire collection were completed. Bone turnover markers (BTMs) including osteocalcin (OC) and beta cross-linked C-telopeptide of type 1 collagen (β-CTX) were tested. Bone mineral density (BMD) of the femoral neck and lumbar vertebrae 1–4 were measured using dual energy X-ray absorptiometry. Fracture risk assessment tool (FRAX) values were calculated to show the probability of major osteoporotic fracture (PMOF) and probability of hip fracture (PHF) over the next 10 years.

**Results:** A total of 1,051 participants were included in this study, including 553 in the urban areas and 498 in the urban-rural fringe areas. The average PMOF and PHF were 3.4 (2.3–5.4) % and .6 (.3–1.5) %, respectively. Compared with that in the urban populations, the femoral neck BMD in the urban-rural fringe populations was lower and FRAX values were generally higher, especially for women. FRAX values in various populations were mainly negatively correlated with lumbar and femoral neck BMD and were positively correlated with *β*-CTX; meanwhile, only PHF was negatively correlated with OC. After adjusting for sex, elevated *β*-CTX levels significantly increased the risk of high PMOF in various populations and increased the risk of high PHF in the urban-rural fringe populations. In particular, the risks of increased PMOF and PHF could increase by as much as 33 times and 19.5 times, respectively, in the urban-rural fringe areas.

**Conclusion:** The urban-rural fringe populations in Southern China may be at risk of osteoporotic fracture. In addition to being related to BMD, the FRAX value also correlates with some BTMs. Combining FRAX with BMD, and BTMs may better predict the fracture risk.

## Introduction

One osteoporotic fracture occurs every 3 s worldwide. The latest epidemiological results on mainland China have revealed that, in the population aged ≥40 years, the prevalence of osteoporosis was 5.0% among men and 20.6% among women, and the prevalence of vertebral fractures was 10.5% and 9.7% among men and women, respectively. In the past 5 years, the prevalence of clinical fractures was 4.1% among men and 4.2% among women (Wang et al., 2021). These findings suggest that prevention of osteoporotic fractures should be emphasized at present. The fracture risk assessment tool (FRAX), recommended by the World Health Organization, has become popular worldwide. The FRAX is a computer-based algorithm that calculates the probability of a major osteoporotic fracture (PMOF) and the probability of a hip fracture (PHF) over the next 10 years (John et al., 2018). PMOF mainly includes clinical spine, forearm, hip or shoulder fractures. FRAX is most commonly used in patients with at least one clinical risk factor for osteoporotic fractures and is especially suitable for those who have never had a fracture in the past but have reduced bone mass.

However, the FRAX algorithm has several limitations. For example, it is only applicable to people aged 40–90 years and is not suitable for those who have been diagnosed with osteoporosis or treated for it, or patients suffering from fragility fractures. However, one of the advantages of FRAX is that the femoral neck bone mineral density (BMD) can be optionally input into the calculation model. This enables the FRAX to be used as a simple or self-screening tool for the general population. Therefore, it can be widely used in families, communities, or primary medical centers where BMD testing conditions are not available. In most areas, a PMOF ≥20% or PHF ≥3% indicates a high risk of osteoporotic fracture, which can be used as intervention thresholds to initiate anti-osteoporosis treatment. Theses thresholds are also recommended by the Diagnosis and Treatment Guidelines for Osteoporosis in China (Chinese Society of Osteoporosis and Bone Mineral Research, 2017).

Osteoporosis and osteoporotic fractures are associated with many environmental factors. With rapid urbanization in China, the living conditions and quality of life of citizens have greatly improved. It is worth noting that people’s physical activity has gradually decreased, likely due to the change from heavy farm work to easy housework, and even a sedentary lifestyle. Changes in lifestyle inevitably lead to subtle changes in the characteristics of osteoporosis and its fractures. This cross-sectional study was conducted in two districts of Nanchang City, Jiangxi Province, Southern China. First, the Xihu District is located in the central area of Nanchang City. Urban residents have stable jobs and incomes, limited physical activities, complete social medical security, and rampant chronic diseases. Second, Qingshanhu District is located in an urban-rural fringe area. With the expansion of the urban scale in recent years, residents in the urban-rural fringe are no longer engaged in agricultural labor, and their lifestyle has changed dramatically, thus leading to an increase in chronic diseases.

Based on the difference between the urban populations and urban-rural fringe populations, this study aimed to compare the application characteristics of FRAX in the two populations, and add BMD and bone turnover markers (BTMs) for analysis, in order to better predict fracture risk.

## Materials and methods

The study was conducted in accordance with the Declaration of Helsinki (revised in 2013). The study protocol was approved by the ethics committee of the Third Hospital of Nanchang. Written informed consent was obtained from all the participants. This study is from the cooperation project of the International Osteoporosis Foundation (IOF) in China (IOFCJO-D001).

### Study design

This cross-sectional study was conducted in June 2018. First, we carried out this study in eight community health service centers (Nanpu, Shizi Street, Shengjinta, and Chaoyang communities in Xihu District, Jingdong Town, Hufang Town, Tangshan town, and Shanghai Road in Qingshanhu District), immediately trained researchers and prepared materials. Second, recruitment methods included posting advertisements on bulletin boards and WeChat groups as well as recruitment *via* telephone appointments. Third, all participants obtained full informed consent in the health service centers. Residents received an appointment form after confirming their identity and signing an informed consent form. Finally, residents provided their informed consent, appointment form, and ID card to the investigation site for registration and health examinations.

### Study population

The inclusion criteria were Han residents, aged 40–90 years old, regardless of sex, who voluntarily participated and signed informed consent. The exclusion criteria included people under the age of 40 or over 90, pregnant or lactating women, people with blood system diseases or X-ray allergy, people who refused any on-site examination, or those who refused to go to the hospital to check BMD. Finally, 1,051 participants were enrolled in the study, including 553 in the Xihu District and 498 in the Qingshanhu District.

### Questionnaire survey

The questionnaire mainly included the clinical risk factors used to calculate the FRAX values, such as name, age, sex, menopausal age of women, previous fracture (yes or no), parent fractured hip (yes or no), current smoking (yes or no), glucocorticoids use (yes or no), rheumatoid arthritis (yes or no), secondary osteoporosis (yes or no), and alcohol consumption of three or more units per day (yes or no).

### Physical examination

Trained researchers measured blood pressure, height, weight, and waist circumference at the survey site. Blood pressure was measured using five electronic sphygmomanometers (Omron, Kyoto, Japan) in a calm sitting position. Three standard measurements were carried out with an interval of at least 1 min, and the average of three measurements was taken for analysis. The height and weight of each participant were measured with calibrated instruments, accurate to .1 cm and .1 kg respectively. Body mass index (BMI) was calculated using the following formula: BMI = weight (kg)/height^2^ (m^2^). Waist circumference was measured with a soft ruler around the abdomen horizontally, accurate to .1 cm.

### Sample testing

Participants were required to take an oral glucose tolerance test (OGTT) on site after 8–10 h of fasting. However, those who had been diagnosed with diabetes or were taking antidiabetic drugs would be exempt from the OGTT. Fasting blood glucose (FBG), two-hour postprandial blood glucose (2hBG), blood urea nitrogen (BUN), serum creatinine (SCr), total cholesterol (CHOL), triglycerides (TG), high-density lipoprotein cholesterol (HDL-C), and low-density lipoprotein cholesterol (LDL-C) levels were measured using the same autoanalyzer (Roche, Basel, Switzerland). Biochemical indicators of bone metabolism were also measured, such as serum calcium (Ca) and phosphorus (P) levels (5,800, Beckman Coulter, California, United States), as well as serum 25-hydroxyvitamin D (25(OH)D), osteocalcin (OC), and beta-cross-linked C-telopeptide of type 1 collagen (β-CTX) concentrations (COBAS E602, Roche, Basel, Switzerland). OC and *β*-CTX are BTMs, that reflect bone formation and bone resorption, respectively.

Estimated glomerular filtration rate (eGFR) was calculated using the Chronic Kidney Disease Epidemiology Collaboration equation. According to the 1999 World Health Organization diagnostic criteria, glucose metabolism can be divided into normal glucose tolerance, prediabetes, and diabetes. According to the National Cholesterol Education Program (NCEP) Expert Panel on Detection, Evaluation, and Treatment of High Blood Cholesterol in Adults (Adult Treatment Panel III) criteria, dyslipidemia was defined as CHOL ≥6.22 mmol/L, HDL-C <1.04 mmol/L, LDL-C ≥4.14 mmol/L, and/or TG ≥2.26 mmol/L.

### BMD measurement

According to the appointment time arranged at the investigation site, the participants took their ID card and the appointment form to the Third Hospital of Nanchang for BMD examination, which was completed within 3 months. The BMD of the femoral neck and lumbar vertebrae 1–4 was measured using dual energy X-ray absorptiometry (DXA) (Medilink, Montpellier, France).

### FRAX assessment

By clicking on the website: http://www.shef.ac.uk/FRAX, and then selecting the language Chinese simplified, we entered the FRAX model to evaluate the fracture risk of people. After inputting the 11 clinical risk factors collected in the questionnaire, we selected BMD as “DMS/Medilink” and input the femoral neck BMD in item 12. Finally, on clicking the calculate button, we obtained the values of PMOF and PHF over the next 10 years.

### Statistical analyses

All trained persons input the questionnaires, BMD results, and FRAX values into the EpiData software (Odense, Denmark). After a double-check, it was combined with the downloaded blood test results to form the final database.

All analyses were performed using the SAS 9.1 software (North Carolina, United States). Categorical variables are presented as numbers (proportions). Continuous variables are presented as means ± standard deviations (SD) or medians (interquartile ranges), and all non-normally distributed data were logarithmically transformed. Student’s t-test and chi-squared test were used to compare the means and proportions of residents in the two different districts. Spearman’s correlation analysis and stepwise regression analysis were used to evaluate the correlation between FRAX values, and BMD, and BTMs. Logistic regression analysis was used to assess the effect of BTMs on fracture risks. The difference was statistically significant with *p*-value <.05.

## Results

### Characteristics of population

The mean age of the study population was 59 ± 8 years. Of the participants, 76.2% were women, of which 84.6% were menopausal, and the mean menopausal age was 49 ± 4 years. The prevalence of previous fractures, diabetes, and dyslipidemia were 19.9%, 13.8%, and 29.2%, respectively (Table 1).

**TABLE 1:** Comparison of population characteristics between the urban and urban-rural fringe areas.

Variables	The whole population	The urban populations (1)	The urban-rural fringe populations (2)	*p*-value (1 vs*.* 2)
Numbers	1051	553	498	—
Age (years)	59 ± 8	58 ± 7	61 ± 9	<.0001
Female (n,%)	801 (76.2%)	429 (77.6%)	372 (74.7%)	.25
Postmenopausal women (n,%)	678 (84.6%)	362 (84.4%)	316 (84.9%)	.83
Menopausal age (years)	49 ± 4	49 ± 4	49 ± 4	.10
BMI (kg/m^2^)	24.06 ± 3.14	23.91 ± 3.11	24.24 ± 3.17	.10
Waist circumference (cm)				
Female	81.4 ± 9.3	83.2 ± 8.3	79.4 ± 10.0	<.0001
Male	85.8 ± 9.1	87.2 ± 8.3	84.4 ± 9.8	.02
Systolic pressure (mmHg)	126 (114–142)	126 (113–140)	127 (115–143)	.11
Diastolic pressure (mmHg)	78 ± 11	78 ± 11	78 ± 11	.30
Current smoking (n, %)	103 (10.4%)	48 (9.0%)	55 (12.1%)	.25
Alcohol consumption of three or more units per day (n, %)	136 (13.8%)	83 (15.5%)	53 (11.7%)	.007
Previous fracture (n, %)	209 (19.9%)	99 (17.9%)	110 (22.1%)	.09
Parent fractured hip (n, %)	115 (10.9%)	64 (11.6%)	51 (10.2%)	.49
Glucocorticoids use (n, %)	18 (1.7%)	9 (1.63%)	9 (1.81%)	.82
Rheumatoid arthritis (n, %)	91 (8.7%)	35 (6.3%)	56 (11.2%)	.005
Secondary osteoporosis (n, %)	36 (3.4%)	15 (2.7%)	21 (4.2%)	.18
FBG (mmol/L)	5.40 (4.96–5.90)	5.60 (5.28–6.07)	5.09 (4.65–5.60)	<.0001
2hBG (mmol/L)	6.74 (5.70–8.15)	6.74 (5.74–7.98)	6.73 (5.49–8.35)	.27
Glucose metabolism (n, %)				.82
Normal glucose tolerance	651 (61.9%)	341 (66.6%)	310 (62.25%)	
Prediabetes	255 (24.3%)	138 (25.0%)	117 (23.5%)	
Diabetes	145 (13.8%)	74 (13.4%)	71 (14.3%)	
BUN (mmol/L)	4.84 (4.07–5.76)	4.79 (4.10–5.72)	4.87 (4.07–5.79)	.63
SCr (μmol/L)	58 (49–68)	52 (45–61)	63 (57–75)	<.0001
eGFR (mL/min/1.73 m^2^)	129 (110–154)	147 (126–171)	115 (101–129)	<.0001
eGFR <90 mL/min/1.73 m^2^ (n, %)	72 (7.3%)	18 (3.4%)	54 (11.9%)	<.0001
CHOL (mmol/L)	5.07 (4.48–5.70)	5.32 (4.68–5.97)	4.87 (4.29–5.41)	<.0001
HDL-C (mmol/L)	1.49 ± .36	1.55 ± .41	1.41 ± .29	<.0001
LDL-C (mmol/L)	2.91 (2.48–3.38)	2.98 (2.55–3.55)	2.80 (2.38–3.25)	<.0001
TG (mmol/L)	1.37 (.97–1.95)	1.33 (.94–1.93)	1.42 (1.01–1.96)	.05
Dyslipidemia (n, %)	302 (29.2%)	168 (31.3%)	134 (27.0%)	.13

BMI, body mass index; FBG, fasting blood glucose; 2hBG, two-hour postprandial blood glucose; BUN, blood urea nitrogen; SCr, serum creatinine; eGFR, estimated glomerular filtration rate; CHOL, total cholesterol; HDL-C, high-density lipoprotein cholesterol; LDL-C, low-density lipoprotein cholesterol; TG, triglycerides.

Compared with the urban-rural fringe populations, the urban populations had a larger waist circumference (both in men and women), and more participants consumed three or more units of alcohol daily.

There were no differences in glucose metabolism and lipid metabolism; however, more people in the urban-rural fringe had decreased eGFR (<90 mL/min/1.73 m^2^) (Table 1).

### Comparison of BMD and FRAX values

The mean BMD of lumbar vertebrae 1–4 in all participants was .78 ± .15 g/cm^2^ and that of the femoral neck was .77 ± .13 g/cm^2^. The average PMOF and PHF were 3.4 (2.3–5.4)% and .6 (.3–1.5)%, respectively.

There was no sex difference in the lumbar BMD between the two districts. The femoral neck BMD of urban-rural fringe females was lower than that of urban females (urban female: .76 ± .12 g/cm^2^, urban-rural fringe female: .73 ± .12 g/cm^2^, *p* = .002), however, there was no difference in males (Table 2).

**TABLE 2:** Comparison of BMD and FRAX values between the urban and urban-rural fringe females and males.

Variables		The urban populations	The urban-rural fringe populations	*p*-value
Lumbar BMD (g/cm^2^)	All	.78 ± .15	.80 ± .15	.05
	Female	.76 ± .14	.77 ± .15	.10
	Male	.85 ± .15	.87 ± .15	.42
femoral neck BMD (g/cm^2^)	All	.78 ± .13	.75 ± .13	.0008
	Female	.76 ± .12	.73 ± .12	.002
	Male	.82 ± .15	.79 ± .13	.10
PMOF (%)	All	3.3 (2.3–5.0)	3.6 (2.5–6.1)	.002
	Female	3.4 (2.4–5.1)	4.0 (2.6–6.7)	.0003
	Male	2.9 (1.9–4.7)	2.9 (1.9–4.3)	.72
PHF (%)	All	.6 (.3–1.2)	.8 (.3–1.9)	<.0001
	Female	.5 (.2–1.2)	.7 (.3–2.0)	<.0001
	Male	.7 (.3–1.7)	.9 (.4–1.6)	.44

BMD, bone mineral density; FRAX, fracture risk assessment tool; PMOF, probability of a major osteoporotic fracture; PHF, probability of hip fracture; Lumbar BMD, was the BMD, of lumbar vertebrae 1–4.

It was found that the 10-year probabilities of fractures in the urban-rural fringe areas were generally higher than those in the urban areas. However, these differences were mainly observed in females. The PMOF was 3.4 (2.4–5.1)% in urban females and 4.0 (2.6–6.7)% in urban-rural fringe females (*p* = .0003), and the PHF was .5 (.2–1.2)% in urban females and .7 (.3–2.0)% in urban-rural fringe females (*p* < .0001), respectively (Table 2).

### Comparison of biochemical indicators of bone metabolism

As a bone metabolism regulating hormone, the mean level of 25(OH)D in the whole population was 54.63 ± 16.74 nmol/L. Serum Ca and P, both biochemical markers, were 2.31 (2.23–2.38) and 1.04 (.92–1.15) mmol/L, respectively. The average levels of OC and *β*-CTX were 11.20 (9.00–14.60) and .22 (.16–.31) ng/mL, respectively.

In female participants, higher 25(OH)D and lower serum P concentrations were found in the urban-rural fringe areas than those in the urban areas. For both males and females, the levels of serum Ca and the bone resorption marker *β*-CTX in the urban-rural fringe were lower than those in the urban areas. As a marker of bone formation, OC showed no difference between the two different areas (Table 3).

**TABLE 3:** Comparison of biochemical indicators of bone metabolism between the urban and urban-rural fringe females and males.

Variables		The urban populations	The urban-rural fringe populations	*p*-value
25(OH)D (nmol/L)	All	52.60 ± 15.55	56.78 ± 17.69	<.0001
	Female	50.24 ± 14.26	53.95 ± 15.21	.0006
	Male	60.38 ± 17.08	65.17 ± 21.52	.06
Ca (mmol/L)	All	2.34 (2.28–2.40)	2.27 (2.15–2.35)	<.0001
	Female	2.34 (2.28–2.40)	2.27 (2.16–2.35)	<.0001
	Male	2.33 (2.27–2.39)	2.27 (2.14–2.35)	<.0001
P (mmol/L)	All	1.05 (.94–1.16)	1.03 (.90–1.13)	.04
	Female	1.08 (.98–1.18)	1.06 (.94–1.14)	.04
	Male	.96 (.85–1.05)	.92 (.83–1.07)	.82
OC (ng/mL)	All	11.20 (9.10–14.70)	14.40 (11.30–18.35)	.46
	Female	12.20 (9.60–15.1)	11.90 (9.40–15.20)	.40
	Male	9.55 (8.00–11.60)	9.90 (7.90–11.90)	.74
β-CTX (ng/mL)	All	.24 (.16–.34)	.20 (.15–.27)	<.0001
	Female	.24 (.17–.35)	.21 (.15–.27)	<.0001
	Male	.22 (.16–.33)	.18 (.14–.25)	.0004

25(OH)D, 25-hydroxyvitamin D; Ca, calcium; P, phosphorus; OC, osteocalcin; *β*-CTX, beta cross-linked C-telopeptide of type 1 collagen. 25(OH)D was bone metabolism regulating hormone, Ca and P were biochemical markers, OC, and *β*-CTX, were bone turnover markers.

### Correlation of FRAX values with BMD and BTMs

Spearman’s correlation analysis and stepwise regression analysis were performed, with PMOF and PHF as dependent variables and lumbar BMD, femoral neck BMD, 25(OH)D, Ca, P, OC, and *β*-CTX as independent variables.

The results in the whole population suggested that PMOF was negative correlated with lumbar and femoral neck BMD and serum Ca levels and was positively correlated with *β*-CTX. Meanwhile, PHF was negatively correlated with lumbar and femoral neck BMD, Ca, P, and OC, and positively correlated with 25(OH)D and *β*-CTX (Table 4).

**TABLE 4:** Spearman’s correlation and stepwise regression analysis of BMD and BTMs associated with FRAX values in the whole population.

Variables	Log PMOF				Log PHF			
	r	*p*-value	β	*p*-value	r	*p*-value	β	*p*-value
Lumbar BMD	−.92881	<.0001	−.24909	<.0001	−1.77831	<.0001	−.20207	.02
femoral neck BMD	−1.40352	<.0001	−1.14035	<.0001	−3.46985	<.0001	−3.35254	<.0001
25(OH)D	−.00106	.03			.00025184	.79	.0028	<.0001
Log Ca	−.50715	.049	−.6541	.0008	−.63647	.20	−.80704	.01
Log P	.26713	.02			−.09657	.22	−.4259	.003
Log OC	.24826	<.0001			.28631	.005	−.28767	.0003
Log *β*-CTX	.20441	<.0001	.1078	.0007	.3295	<.0001	.28542	<.0001

In urban residents, it was verified that PMOF was still negatively correlated with lumbar and femoral neck BMD and was positively correlated with *β*-CTX. PHF was mostly negatively correlated with femoral neck BMD, P, and OC and was positively correlated with 25(OH)D and *β*-CTX (Table 5).

**TABLE 5:** Spearman’s correlation and stepwise regression analysis of BMD and BTMs associated with FRAX values in the urban populations.

Variables	Log PMOF				Log PHF			
	r	*p*-value	β	*p*-value	r	*p*-value	β	*p*-value
Lumbar BMD	−.87381	<.0001	−.17321	.01	−1.78078	<.0001		
femoral neck BMD	−1.29793	<.0001	−1.12035	<.0001	−3.47346	<.0001	−3.47492	<.0001
25(OH)D	−.00062731	.37			.00020021	.88	.00234	.002
Log Ca	−.09876	.88			−.69853	.58		
Log P	.36015	.02			−.13979	.64	−.55791	.001
Log OC	.36784	<.0001			.46207	.001	−.25806	.009
Log *β*-CTX	.23765	<.0001	.0734	.04	.39484	<.0001	.2746	<.0001

In the urban-rural fringe areas, we found that PMOF was negatively correlated with BMD and Ca and was positively correlated with *β*-CTX. PHF was negatively correlated with BMD and OCwas positively correlated with *β*-CTX (Table 6).

**TABLE 6:** Spearman’s correlation and stepwise regression analysis of BMD and BTMs associated with FRAX values in the urban-rural fringe populations.

Variables	Log PMOF				Log PHF			
	r	*p*-value	β	*p*-value	r	*p*-value	β	*p*-value
Lumbar BMD	−1.04519	<.0001	−.39863	<.0001	−1.88396	<.0001	−.33024	.02
femoral neck BMD	−1.55624	<.0001	−1.18325	<.0001	−3.42995	<.0001	−3.25238	<.0001
25(OH)D	−.00174	.01			−.00041299	.75		
Log Ca	−.38825	.22	−.74778	.002	−.03454	.95		
Log P	.21618	.19			.03064	.92		
Log OC	.14386	.06			.16162	.26	−.45505	.0007
Log *β*-CTX	.22383	.0004	.21629	.0005	.39610	.001	.4088	.0003

In summary, FRAX values in various populations were mainly negatively correlated with lumbar and femoral neck BMD and were positively correlated with *β*-CTX. In addition, PHF and OC were negatively correlated in all participants.

### Effect of BTMs on fracture risks

Further logistic regression analysis was performed to evaluate the effect of BTMs on FRAX values. The dependent variable was increased fracture risk. However, only .4% of the participants reached the threshold level of PMOF ≥20%, and 10.7% reached a PHF ≥3%. Instead, we uniformly defined the increased risk as the FRAX values reaching the upper 1/3 of PMOF or PHF. The independent variables were 25(OH)D, Ca, P, OC, and *β*-CTX levels. Based on the different prevalence and characteristics of osteoporosis in men and women, all the models were adjusted for sex.

First, the thresholds of increased PMOF were higher than 4.6% in the whole population, higher than 4.2% in the urban areas, and higher than 5.0% in the urban-rural fringe areas. In various populations, elevated *β*-CTX levels significantly increased the risk of major osteoporotic fractures, and even increased the risk by up to 33 times in the urban-rural fringe areas [odds ratios (ORs), 95% confidence intervals (CIs) = 3.976 (1.066–14.831), 5.802 (1.071–31.444), and 34.024 (2.642–438.133) in the whole population and urban and urban-rural fringe populations, respectively]. However, OC levels had no statistical effect on high PMOF. In addition, among other markers, only elevated 25(OH)D levels slightly reduced the risk of high PMOF by 1%–1.6% in the whole population and urban-rural fringe populations (Table 7).

**TABLE 7:** Odds ratios for having increased PMOF by different BTMs after adjusting for sex.

Variables	Increased PMOF					
	The whole population		The urban populations		The urban-rural fringe populations	
	OR (95% CI)	*p*-value	OR (95% CI)	*p*-value	OR (95% CI)	*p*-value
25(OH)D	.990 (.981–.999)	.04	.990 (.977–1.003)	.12	.984 (.970–.998)	.03
Ca	.603 (.334–1.089)	.09	.157 (.017–1.415)	.10	2.093 (.901–4.860)	.09
P	1.533 (.525–3.759)	.35	1.891 (.524–6.827)	.33	.442 (.116–1.685)	.23
OC	1.026 (.989–1.065)	.16	1.018 (.966–1.073)	.51	.976 (.925–1.030)	.38
β-CTX	3.976 (1.066–14.831)	.04	5.802 (1.071–31.444)	.04	34.024 (2.642–438.133)	.007

OR, odds ratio; CI, confidence interval. The thresholds of increased PMOF, were higher than 4.6% in the whole population, higher than 4.2% in the urban populations, and higher than 5.0% in the urban-rural fringe populations. All models were adjusted for sex.

Moreover, the thresholds of increased PHF were higher than 1.1% in the whole population, higher than .9% in the urban areas, and higher than 1.4% in the urban-rural fringe areas. After adjustment for sex, *β*-CTX was no longer significant for high PHF in the overall and urban populations. However, it is worth mentioning that the increase in *β*-CTX dramatically increased this risk by nearly 19.5 times in the urban-rural fringe areas [OR (95% CI) = 20.484 (1.228–16.471)], suggesting that *β*-CTX is extremely important for the early warning of this population (Table 8).

**TABLE 8:** Odds ratios for having increased PHF by different BTMs after adjusting for sex.

Variables	Increased PHF					
	The whole population		The urban populations		The urban-rural fringe populations	
	OR (95% CI)	*p*-value	OR (95% CI)	*p*-value	OR (95% CI)	*p*-value
25(OH)D	.994 (.986–1.003)	.22	1.002 (.989–1.015)	.78	.989 (.976–1.022)	.11
Ca	.707 (.392–1.274)	.25	.234 (.026–2.143)	.20	1.133 (.519–2.473)	.75
P	1.085 (.443–2.653)	.86	1.322 (.358–4.880)	.68	.805 (.221–2.935)	.74
OC	1.027 (.990–1.065)	.16	1.060 (1.005–1.118)	.03	.989 (.938–1.044)	.69
β-CTX	3.685 (.994–13.654)	.05	4.904 (.906–26.553)	.07	20.484 (1.707–245.826)	.02

The thresholds of increased PHF, were higher than 1.1% in the whole population, higher than .9% in the urban populations, and higher than 1.4% in the urban-rural fringe populations. All models were adjusted for sex.

## Discussion

According to the degree of BMD reduction, low bone mass is usually defined as 1–2.5 SD lower than the average peak bone mass of healthy adults of the same sex and race. There are many people with low bone mass in China, who fall into the high-risk group for the development of osteoporosis. Epidemiological data revealed that the prevalence of low bone mass in China is 32.9% in people aged 40–49 years, including 31.2% in the urban areas and 33.9% in the rural areas, and is 46.4% in people over 50 years of age, including 45.4% in the urban areas and 46.9% in the rural areas (Chinese Society of Osteoporosis and Bone Mineral Research, 2019). This report suggests that there are more people with low bone mass in rural China, and the risk of osteoporosis tends to be higher. The latest survey confirms this finding (Wang et al., 2021). The prevalence of osteoporosis was higher in the rural areas (22.3%) than that in the urban areas (17.3%) (*p* <.001) among females, and the prevalence of vertebral fracture was significantly higher among males in the rural areas (11.8%) than that in the urban areas (8.2%) (*p* = .008). In the past 5 years, the prevalence of clinical fractures in the urban and rural areas was similar, both among males and females. In addition, rural residence has become a new risk factor (other factors include well-known factors such as female sex, older age, lower BMI, smoking, etc.,), which is significantly associated with lower BMD in the lumbar spine (L1 to L4). With the promotion of new rural construction and urban expansion, the urban-rural fringe areas have become a new focus, with particular interest being paid to the disease characteristics of the population. In other words, with a sharp decline in physical activity, a gradually urbanized lifestyle, and a fast-growing economy, residents in the urban-rural fringe areas are more likely to suffer from various chronic non-communicable diseases without knowing.

Consistent with the above epidemiological findings, this study showed that the average femoral neck BMD of urban-rural fringe residents was lower than that of urban residents, suggesting that the urban-rural fringe populations, especially females, might be a potential contributor to osteoporosis. According to the comparison between the groups, central obesity, alcohol consumption, high FBG and cholesterol levels were more common in the urban areas, and people in the urban-rural fringe areas seemed to be healthier. However, in the case of no difference in fracture history between the groups, bone and joint discomfort was more obvious in the urban-rural fringe populations (such as more self-reported rheumatoid arthritis), which indirectly reflected that they might be in the stage of low bone mass or pre-osteoporosis. However, this is insufficient. We did not collect information on diets rich in calcium and vitamin D supplementation, outdoor activity time, or frequency of falls, resulting in no comparison of more osteoporosis risk factors between the two groups. In addition, these two results deserve public attention. First, the calculated probability of osteoporotic fracture in the urban-rural fringe participants (mainly females) was generally higher than that of urban residents. It is urgent to strengthen the early screening and intervention of osteoporosis in the urban-rural fringe areas and to improve the prevention and control of osteoporosis in primary health institutions. Second, our results showed that FRAX values were generally at a relatively low level in the general population, and the number of people with PMOF ≥20% or PHF ≥3% was very small. Does this mean that our population is not at a high risk of osteoporosis fractures? Alternatively, does this mean that the FRAX value has no significance in predicting fractures? Probably not. Although affected by multiple comorbidities, FRAX can significantly discriminate the fracture risk of non-dialysis patients with chronic kidney disease, especially major osteoporotic fractures (Whitlock et al., 2019). In another study (Wu et al., 2022), 51.8% of 164 hemodialysis patients had a high risk of fractures according to the FRAX threshold. It was also found that a high risk of fractures based on FRAX was independently associated with all-cause mortality. However, the FRAX thresholds are not compatible between countries and regions. FRAX is also believed to underestimate the fracture probability in some cases, such as in individuals with prior fractures (Wu et al., 2014). Therefore, more factors need to be combined with FRAX to assess the risk. On the one hand, we defined the upper 1/3 of the FRAX value as a high risk of fracture, and on the other hand, we integrated BMD and BTMs into fracture risk assessment.

Stepwise regression analysis confirmed that FRAX fracture probability was negatively correlated not only with femoral neck BMD (included in the algorithm) but also with lumbar BMD. Therefore, BMD of any traditional load-bearing bone should be considered. At the same time, the FRAX value combined with BMD is superior to clinical risk factors or isolated BMD in predicting fractures (Wu et al., 2014), therefore, it may be prudent to combine the BMD of the lumbar vertebrae and femoral neck with the FRAX calculation. In addition, BTMs are another osteoporosis factors used in this study, including the bone formation marker OC and the bone resorption marker *β*-CTX. Stepwise regression analysis revealed that FRAX fracture probability was positively correlated with *β*-CTX, and PHF was additionally negatively correlated with OC in different populations. Through logistic regression analysis, we further observed that the increase in *β*-CTX levels significantly increased the risk of high PMOF in all models and increased the risk of high PHF in the urban-rural fringe populations, regardless of sex. No effect of OC was observed. At present, BTMs are not included in the FRAX algorithm, and official claims that BTMs predict fractures independently of BMD are uncertain. It was found to be independent in some studies (Gerdhem et al., 2004; Meier et al., 2005; Yoon and Yu, 2018), but not in all studies (Garnero et al., 2002; Bauer et al., 2009; Vilaca et al., 2017; Han et al., 2022). There is an inherent negative correlation between BTMs and BMD (either the femoral neck or lumbar vertebrae BMD). With the increase in age and the decline in estrogen levels in postmenopausal women, bone turnover becomes stronger (Jia and Cheng, 2022). Our study also found that BTMs and BMD could enter the multiple stepwise regression equation and had an impact on FRAX fracture risk. However, we did not observe any effect of BTMs independently of BMD. Therefore, whether the association between BTMs and fracture risk is affected by BMD cannot be determined, and BTMs cannot be included in the FRAX calculation as a risk factor (McCloskey et al., 2011).

Nevertheless, International Osteoporosis Foundation and International Federation of Clinical Chemistry and Laboratory Medicine (IOF-IFCC) points out that BTMs, especially bone resorption markers, have a certain practicability in predicting fractures. However, it is difficult to draw a clear conclusion because various BTMs were measured, different fracture sites were included, and the groupings of BTMs were inconsistent in previous studies (Vasikaran et al., 2011). Our study also found that the correlation analysis results of multiple biochemical indicators of bone metabolism were inconsistent. Compared with bone formation markers and other biochemical indicators, the correlation between the bone resorption marker *β*-CTX and fracture risk was clearer, which was consistent with the results of previous studies (Vasikaran et al., 2011). In addition, elevated *β*-CTX levels could significantly increase the risk of osteoporotic fractures by nearly 20–35 times in the urban-rural fringe areas, although the average *β*-CTX levels in males and females in this area were not high. These figures suggest that the increase in *β*-CTX would make the urban-rural fringe populations extremely vulnerable to osteoporosis and fractures. Therefore, after screening high-risk participants for fractures by FRAX, attention should be paid to the examination of *β*-CTX and BMD, which will contribute to the timeliness and effectiveness of early intervention.

In our study, OC was negatively correlated with PHF in all populations; however, we did not identify the effect of OC levels on the high fracture risk. IOF-IFCC recommends serum type I procollagen amino-terminal peptide (s-PINP) as the preferred bone formation marker (Vasikaran et al., 2011); however, OC is not only a bone formation marker but also an important metabolically active hormone involved in regulating glucose and lipid metabolism (Fukumoto and Martin, 2009; Magni et al., 2016; Dirckx et al., 2019; Cipriani et al., 2020). Recent study found that low OC levels in circulation increase the risk of incident diabetes and diabetic kidney disease (DKD) (Ye et al., 2022). As a representative metabolic disease, diabetes is a prominent risk factor for osteoporosis and related fractures. Therefore, some scholars believe that diabetes is not sufficiently included in the FRAX algorithm (Chinese Society of Osteoporosis and Bone Mineral Research, 2017). In brief, OC is negatively correlated with PHF (although not strong enough) and also plays an endocrine role of bone in metabolic diseases (Ye et al., 2022); therefore, it can be hypothesized that low OC levels may generate additional predictive values under dual exposure.

In summary, IOF-IFCC recognizes the role of BTMs in the management of osteoporosis and believes that BTMs have good application prospects for fracture prediction and treatment monitoring (McCloskey et al., 2011). However, the popularity of BTMs testing in China is not sufficient and may become an area of interest in the future. In addition, our current evidence also suggests that it is necessary to combine FRAX with BMD and BTMs for a more ideal fracture prediction. The main limitation was the insufficient number of males recruited for the study, which may be due to the relatively low prevalence of osteoporosis in the male population, leading to their unwillingness to complete BMD measurements in hospital. Therefore, the difference in various parameters among men may not be accurately observed because of the insufficient proportions.

## Conclusion

In this study, the probability of a major osteoporotic fracture and hip fracture in the urban-rural fringe populations (especially females) within 10 years was higher than that in the urban populations, suggesting that urban-rural fringe residents in southern China may be at risk of osteoporosis and related fractures. In addition to being related to BMD, the FRAX value also correlates with some BTMs. For example, OC levels were negatively correlated with the probability of hip fracture and high *β*-CTX levels significantly increased the risk of osteoporotic fractures, especially in the urban-rural fringe populations. Therefore, we need to combine FRAX with BMD and BTMs to better predict the risk of fracture.

## Data Availability

The raw data supporting the conclusion of this article will be made available by the authors, without undue reservation.

## References

[B1] Bauer D. C. Garnero P. Harrison S. L. Cauley J. A. Eastell R. Ensrud K. E. (2009). Biochemical markers of bone turnover, hip bone loss, and fracture in older men: The MrOS study. J. Bone. Min. Res. 24 (12), 2032–2038. 10.1359/jbmr.090526 PMC2791517 19453262

[B2] Chinese Society of Osteoporosis and Bone Mineral Research (2019). Epidemiological results of osteoporosis in China and the results of “healthy bones” special action. Chin. J. Osteoporos. Bone. Min. Res. 12 (3), 317–318. 10.3969/j.issn.1674-2591.2019.04.001

[B3] Chinese Society of Osteoporosis and Bone Mineral Research (2017). Guidelines for diagnosis and management of primary osteoporosis (2017). Chin. J. Osteoporos. 10 (5), 413–443. 10.3969/j.issn.1006-7108.2019.03.001

[B4] Cipriani C. Colangelo L. Santori R. Renella M. Mastrantonio M. Minisola S. (2020). The Interplay between bone and glucose metabolism. Front. Endocrinol. (Lausanne). 11, 122. 10.3389/fendo.2020.00122 32265831 PMC7105593

[B5] Dirckx N. Moorer M. C. Clemens T. L. Riddle R. C. (2019). The role of osteoblasts in energy homeostasis. Nat. Rev. Endocrino. 15 (11), 651–665. 10.1038/s41574-019-0246-y PMC6958555 31462768

[B6] Fukumoto S. Martin T. J. (2009). Bone as an endocrine organ. Trends. Endocrinol. Metab. 20 (5), 230–236. 10.1016/j.tem.2009.02.001 19546009

[B7] Garnero P. Cloos P. Sornay-Rendu E. Qvist P. Delmas P. D. (2002). Type I collagen racemization and isomerization and the risk of fracture in postmenopausal women: The OFELY prospective study. J. Bone. Min. Res. 17 (5), 826–833. 10.1359/jbmr.2002.17.5.826 12009013

[B8] Gerdhem P. Ivaska K. K. Alatalo S. L. Halleen J. M. Hellman J. Isaksson A. (2004). Biochemical markers of bone metabolism and prediction of fracture in elderly women. J. Bone. Min. Res. 19 (3), 386–393. 10.1359/JBMR.0301244 15040826

[B9] Han M. S. Lee G. J. Lee S. K. Lee J. K. Moon B. J. (2022). Clinical application of bone turnover markers in treating osteoporotic vertebral compression fractures and their role in predicting fracture progression. Med. Baltim. 101 (32), e29983. 10.1097/MD.0000000000029983 PMC9371513 35960080

[B10] Jia L. Cheng M. (2022). Correlation analysis between risk factors, BMD and serum osteocalcin, CatheK, PINP, β-crosslaps, TRAP, lipid metabolism and BMI in 128 patients with postmenopausal osteoporotic fractures. Eur. Rev. Med. Pharmacol. Sci. 26, 7955–7959. 10.26355/eurrev_202211_30147 36394744

[B11] John A. K. Helena J. Nicholas C. H. Eugene V. M. (2018). A brief history of FRAX. Arch. Osteoporos. 13 (1), 118. 10.1007/s11657-018-0510-0 30382424 PMC6290984

[B12] Magni P. Macchi C. Sirtori C. R. Romanelli M. M. C. (2016). Osteocalcin as a potential risk biomarker for cardiovascular and metabolic diseases. Clin. Chem. Lab. Med. 54 (10), 1579–1587. 10.1515/cclm-2015-0953 26863345

[B13] McCloskey E. V. Vasikaran S. Cooper C. (2011). Position development conference MembersOfficial positions for FRAX^®^ clinical regarding biochemical markers from joint official positions development conference of the international society for clinical densitometry and international osteoporosis foundation on FRAX^®^ . J. Clin. Densitom. 14 (3), 220–222. 10.1016/j.jocd.2011.05.008 21810528

[B14] Meier C. Nguyen T. V. Center J. R. Seibel M. J. Eisman J. A. (2005). Bone resorption and osteoporotic fractures in elderly men: The dubbo osteoporosis epidemiology study. J. Bone. Min. Res. 20 (4), 579–587. 10.1359/JBMR.041207 15765176

[B15] Vasikaran S. Eastell R. Bruyère O. Foldes A. J. Garnero P. Griesmacher A. (2011). Markers of bone turnover for the prediction of fracture risk and monitoring of osteoporosis treatment: A need for international reference standards. Osteoporos. Int. 22 (2), 391–420. 10.1007/s00198-010-1501-1 21184054

[B16] Vilaca T. Gossiel F. Eastell R. (2017). Bone turnover markers: Use in fracture prediction. J. Clin. Densitom. 20 (3), 346–352. 10.1016/j.jocd.2017.06.020 28716498

[B17] Wang L. Yu W. Yin X. Cui L. Tang S. Jiang N. (2021). Prevalence of osteoporosis and fracture in China: The China osteoporosis prevalence study. JAMA. Netw. Open. 4 (8), e2121106. 10.1001/jamanetworkopen.2021.21106 34398202 PMC8369359

[B18] Whitlock R. H. Leslie W. D. Shaw J. Rigatto C. Thorlacius L. Komenda P. (2019). The Fracture Risk Assessment Tool (FRAX^®^) predicts fracture risk in patients with chronic kidney disease. Kidney. Int. 95 (2), 447–454. 10.1016/j.kint.2018.09.022 30579724

[B19] Wu C. H. McCloskey E. V. Lee J. K. Itabashi A. Prince R. Yu W. (2014). Consensus of official position of IOF/ISCD FRAX initiatives in Asia-Pacific region. J. Clin. Densitom. 17 (1), 150–155. 10.1016/j.jocd.2013.06.002 23916756

[B20] Wu P. Y. Chen S. C. Lin Y. C. Chen P. C. Chuang W. S. Huang Y. C. (2022). Role of fracture risk assessment tool and bone turnover markers in predicting all-cause and cardiovascular mortality in hemodialysis patients. Front. Med. (Lausanne). 9, 891363. 10.3389/fmed.2022.891363 35463031 PMC9021425

[B21] Ye X. Yu R. Jiang F. Hou X. Wei L. Bao Y. (2022). Osteocalcin and risks of incident diabetes and diabetic kidney disease: A 4.6-year prospective cohort study. Diabetes. Care. 45 (4), 830–836. 10.2337/dc21-2113 35090006 PMC9016737

[B22] Yoon B. H. Yu W. (2018). Clinical utility of biochemical marker of bone turnover: Fracture risk prediction and bone healing. J. Bone. Metab. 25 (2), 73–78. 10.11005/jbm.2018.25.2.73 29900156 PMC5995756

